# A Systematic Review of Diagnostic Performance of Circulating MicroRNAs in Colorectal Cancer Detection with a Focus on Early-Onset Colorectal Cancer

**DOI:** 10.3390/ijms25179565

**Published:** 2024-09-03

**Authors:** Adhari AlZaabi, Asem Shalaby

**Affiliations:** 1 Department of Human and Clinical Anatomy, College of Medicine and Health Sciences, Sultan Qaboos University, Muscat 123, Oman; 2 Department of Pathology, College of Medicine and Health Sciences, Sultan Qaboos University, Muscat 123, Oman; a.shalaby@squ.edu.om

**Keywords:** colorectal cancer, early-onset colorectal cancer, miRNA, biomarkers, non-invasive screening, diagnostic performance

## Abstract

The rising incidence and mortality of early-onset colorectal cancer (EOCRC) emphasize the urgent need for effective non-invasive screening. Circulating microRNAs (miRNAs) have emerged as promising biomarkers for cancer detection. This systematic review aims to evaluate the diagnostic performance of circulating miRNAs in detecting colorectal cancer (CRC). A literature search was conducted in PubMed and Scopus. Studies that report sensitivity, specificity, or area under the curve (AUC) for CRC detection by miRNA were included. The miRNA miR-21 was the most frequently studied biomarker, with a varying range of AUC from 0.55 to 0.973 attributed to differences in study populations and methodologies. The miRNAs miR-210 and miR-1246 showed potential diagnostic capacity with miR-1246 achieving an AUC of 0.924, 100% sensitivity, and 80% specificity. The miRNA panels offer improved diagnostic performance compared to individual miRNA. The best performing panel for CRC patients below 50 is miR-211 + miR-25 + TGF-β1 with AUC 0.99 and 100 specificity and 97 sensitivity. Circulating miRNAs hold significant promise as non-invasive biomarkers for CRC screening. However, the variability in diagnostic performance highlights the need for a standardized method and robust validation studies. Future research should focus on large-scale, ethnically diverse cohorts to establish clinically relevant miRNA biomarkers for CRC, particularly in younger populations.

## 1. Introduction

Colorectal cancer (CRC) ranks as the third most frequently diagnosed cancer globally and is the fourth leading cause of cancer-related mortality. Projections indicate that by 2030, the global burden of CRC will escalate by 60%, resulting in over 2.2 million new cases and 1.1 million cancer-related deaths [1]. Based on the projection of a population aging trend, the global population growth and environmental exposure, it is expected that the global number of new CRC cases will reach 3.2 million in 2040 [2]. The survival rate for early-stage CRC is approximately 90.3%, whereas it drops to 12.5% for advanced-stage cases [3]. Current diagnostic methods for CRC, including colonoscopy, computed tomography (CT), stool DNA testing, and fecal occult blood test (FOBT), are effective but are often invasive, uncomfortable, and inconvenient or they lack sensitivity and specificity [4]. To enhance patient outcomes and survival rates, there is a need for a deeper understanding of the CRC microenvironment and the feasibility of non-invasive screening testing.

Recent research has increasingly focused on the potential of microRNAs (miRNAs) as non-invasive biomarkers for CRC. These miRNAs are endogenous, single-stranded, non-coding RNAs, ranging from 19 to 25 nucleotides in length, that regulate gene expression by binding to mRNAs and preventing their translation into proteins. In CRC, miRNAs can act as either oncogenes, promoting cancer development, or tumor suppressors, inhibiting it. They influence critical cellular processes such as proliferation, apoptosis, angiogenesis, and metastasis [5]. Studies have shown that miRNAs regulate human protein-coding genes, contribute to carcinogenesis, and impact tumor growth [6,7]. While typically generated in the nucleus, miRNAs are also present in serum and extracellular environments, suggesting their functional roles in these contexts [5]. Extracellular miRNAs can be released via secretory processes, primarily in extracellular vesicles (exosomes) or through passive leakage from damaged or dying cells [8]. Given their stability in bodily fluids, circulating miRNAs hold promise as biomarkers for early cancer detection and progression monitoring [5,8]. 

This systematic review aims to consolidate current evidence on the diagnostic utility of circulating miRNAs in CRC with a particular focus on early-onset CRC (EOCRC). By evaluating studies that compare miRNA profiles in CRC patients with healthy controls, this review will highlight the clinical relevance and challenges of miRNA-based diagnostics. Understanding the current state of miRNA biomarkers in CRC detection is crucial for advancing personalized medicine and improving early detection strategies, setting the stage for future research in this dynamic field.

## 2. Methods

### 2.1. Search Strategy and Selection

This systematic review adhered to the Preferred Reporting Items for Systematic Reviews and Meta-Analyses (PRISMA) 2020 guidelines. A comprehensive literature search was conducted using PubMed and SCOPUS databases. The search terms included: (marker OR biomarker) AND (serum OR plasma OR blood) AND (diagnosis OR screening) AND (colorectal OR colon OR rectal) AND (cancer OR carcinoma OR neoplasia). Manual searches of the reference lists of included articles were also performed to identify additional relevant studies. The online search was concluded in May 2023.

### 2.2. Study Selection Criteria

All retrieved articles were imported into Rayyan software for systematic review (https://www.rayyan.ai/ (accessed on 19 August 2024)). Duplicates were removed, and relevant articles were screened based on title and abstract. Full-text review confirmed eligibility.

Inclusion Criteria: Studies included in this review were published between 2019 and 2023 and focused on circulating miRNAs as markers for early detection of colorectal cancer in human populations. The studies involved adult patients aged 18 years or older, with both CRC and control groups, and reported diagnostic accuracy parameters such as AUC, sensitivity, and specificity.

Exclusion Criteria: Studies were excluded if the full-text was not available electronically or if they were commentaries, letters, editorials, protocols, guidelines, case reports, or review articles. In vitro studies and studies lacking sufficient outcome data were also excluded.

### 2.3. Data Extraction

Data extraction was conducted by two independent reviewers (A.Z. and A.S.) using a standardized data extraction form. Collected data included the first author, year of publication, specimen used, number of cases and controls, study characteristics, circulating miRNAs investigated, diagnostic accuracy parameters, and outcomes related to early colorectal cancer detection. Discrepancies between reviewers were resolved through consensus or involvement of a third reviewer if necessary. A narrative synthesis of the findings from the included studies was performed, focusing on the diagnostic potential and performance of circulating miRNA markers in detecting colorectal cancer.

### 2.4. Quality Assessment

The quality of the included studies was evaluated by two independent reviewers (A.Z. and A.S.) using the Joanna Briggs Institute (JBI) tools for case-control studies. Specific checklist items used from the JBI tools are detailed in [9]. Discrepancies were resolved through consensus.

## 3. Results and Discussion

### 3.1. Study Selection

**Figure 1:**
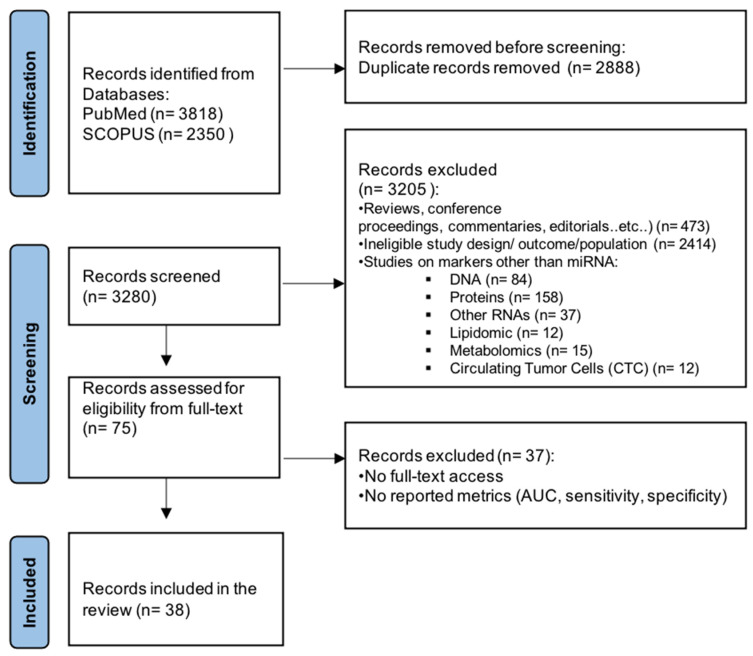
Flowchart of the literature study process and selection.

The flowchart in Figure 1 represents the search and selection strategy for this study. The initial search resulted in a total of 6168 studies, consisting of PubMed (n = 3818) and SCOPUS (n = 2350) articles. After the removal of duplicates (n = 2888) and applying exclusion criteria, 3205 studies were excluded, resulting in 75 articles selected for further evaluation. Additionally, 37 articles were excluded due to absence of reported metrics (AUC, sensitivity, specificity) or lack of full-text access, leaving 38 studies for inclusion in this systematic review.

### 3.2. Study Quality Assessment Graph

**Figure 2:**
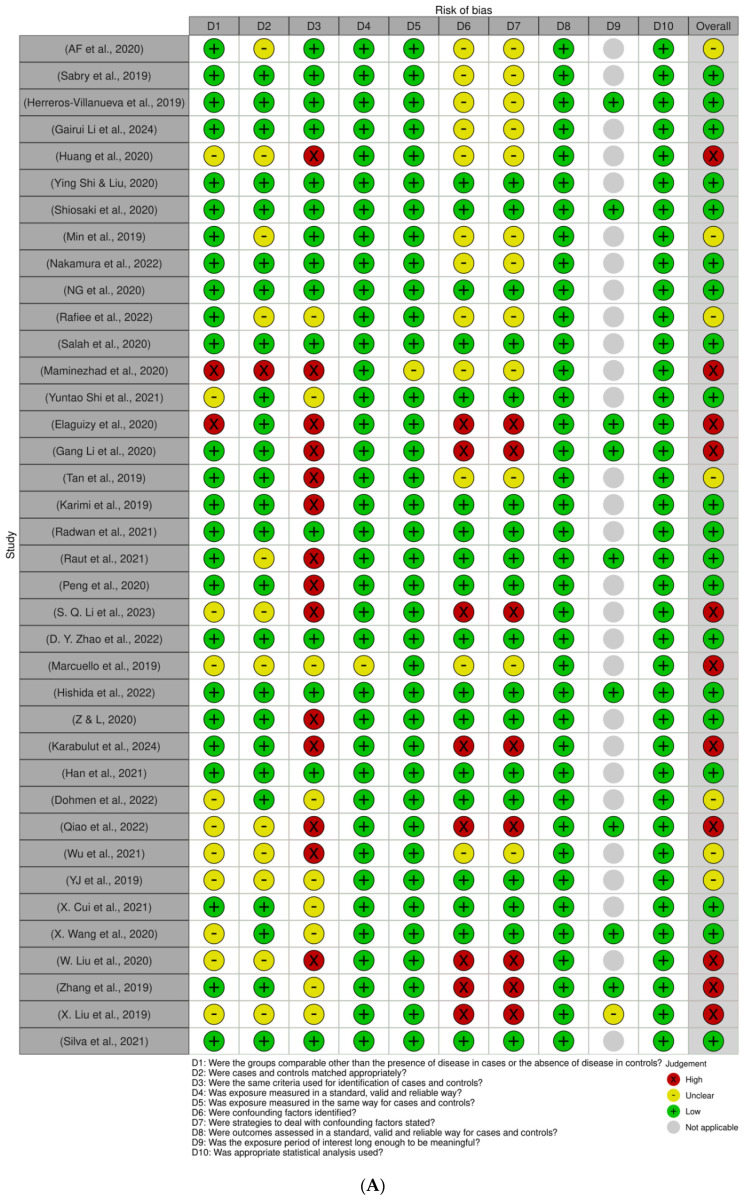

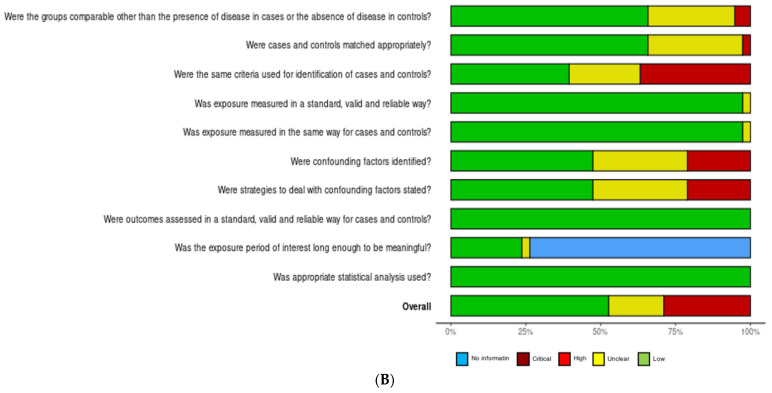
JBI assessment of studies [10,11,12,13,14,15,16,17,18,19,20,21,22,23,24,25,26,27,28,29,30,31,32,33,34,35,36,37,38,39,40,41,42,43,44,45,46,47,48] using ROBvis software (https://www.riskofbias.info/welcome/robvis-visualization-tool, accessed on 19 August 2024). (**A**) graph and (**B**) summary.

The methodological quality of the included studies was evaluated independently by two authors using the JBI checklist. Each item was rated as “yes” (low risk of bias), “no” (high risk of bias), or “unclear”. Disagreements were resolved by consensus. Overall, the quality of the included studies was satisfactory and eligible for review as shown in Figure 2.

### 3.3. Characteristics of the Selected Studies

The 38 included studies utilized a case-control design and were published between 2019 and May 2023. The studies included 2408 cancer cases, 503 colorectal adenomas, and 1950 healthy controls. Sample sizes ranged from 7 to 165 CRC patients. Most studies (20 out of 38) included early-onset colorectal cancer (EOCRC) patients. Serum was the most common sample type used followed by plasma. Thirty-seven studies used quantitative real-time PCR (RT-qPCR) to detect miRNA expression levels, with one study employing sequencing [30]. Among the thirty-eight articles, seventeen focused on single miRNAs, thirteen on panels, and eight on both. Sensitivity of single miRNAs ranged from 32.1% to 100%, specificity from 40% to 100%, and AUC from 0.55 to 0.973. For panel markers, the sensitivity ranged from 21.7% to 100%, specificity from 56% to 100%, and AUC from 0.638 to 0.993.

Table 1 summarizes the study features, noting that several studies only reported AUC values without sensitivity and specificity. Nineteen studies included validation cohorts, either through independent sample sets or different analytical techniques, confirming the clinical utility of identified miRNA markers.

Table 2 shows that the highest AUC reported for a single miRNA marker is 0.973 for miR-21 [12]. Conversely, it was also reported to be the miRNA with the lowest AUC (0.55) along with miRNA-34 which has AUC 0.55 [39]. For panel markers, the highest AUC is 0.993 for a combination of miRNAs (miR-211, miR-25, and TGF-β1) [29] and the lowest AUC for a panel is 0.6385 for a combination of miR-21, miR-29a, and miR-92a [17], suggesting less reliable diagnostic capability. The single miRNA with the highest specificity was miR-627-5p (100%), and the panel that combined miR-627-5p + miR-199a-5p + CEA + CA19 yielded the highest specificity (100%) [33]. 

Eighteen single miRNAs showed AUC values above 0.7, demonstrating significant potential as biomarkers for colorectal cancer detection. Table 3 details only those miRNAs with AUC above 0.7 with their corresponding sensitivity and specificity values reported. The miRNA **miR-21** exhibited the highest AUC of 0.973 [12], with a sensitivity of 91.4% and s specificity of 95%. The miRNA **miR-1290** was reported to have an AUC of 0.96 with a sensitivity of 78.79% and a specificity of 93.33% [47]. The miRNAs **miR-1246** and **miR-378e** both demonstrated high AUC values of 0.924 and 0.9298, respectively, with miR-1246 achieving 100% sensitivity and 73% specificity [22]. Other notable miRNAs include **miR-210** with an AUC of 0.934 [12] and **miR-92a-1** with an AUC of 0.914 [16]. The variability in sensitivity and specificity across different studies underscores the need for further validation and potential combination with other biomarkers for enhanced diagnostics.

Table 4 presents miRNA panels with AUC values exceeding 0.7 with reported sensitivity and specificity, further demonstrating their potential as comprehensive biomarkers for colorectal cancer diagnosis. The inclusion of multiple miRNAs in panels typically enhances diagnostic accuracy. The panel comprising **miR-1290 and miR-320d** achieved an impressive AUC of 0.98, with a sensitivity of 90.91% and a specificity of 93.33% [47]. Similarly, the panel including **miR-627-5p, miR-199a-5p, CEA, and CA19** also reported an AUC of 0.98, with sensitivity and specificity of 98% and 100%, respectively [33]. The combination of **miR-211, miR-25, and TGF-β1** demonstrated the highest AUC of 0.993, with sensitivity and specificity 100% and 97%, respectively [29]. Multiple panels, including those with **miR-19a, miR-19b, miR-15b, miR-29a, miR-335, and miR-18a**, exhibited AUC values around 0.95, reinforcing their diagnostic potential [13].

### 3.4. miRNAs for EOCRC Detection

Table 5 summarizes the performance of single and panel miRNA markers for detecting early-onset colorectal cancer (EOCRC). Among these markers, miR-21 demonstrated the highest diagnostic performance with an AUC of 0.973, a sensitivity of 91.4%, and a specificity of 95% [12]. Additional studies, such as [11], also highlighted miR-21’s strong performance with an AUC of 0.94, a sensitivity of 95.8%, and a specificity of 91.7%. Other notable single miRNA markers included miR-210 with an AUC of 0.934, and miR-144-3p with an AUC of 0.954 [27].

Among the panel markers, the combination of miR-211, miR-25, and TGF-β1 merged as the best-performing panel, achieving an AUC of 0.993 with a sensitivity and a specificity of 97% and 100%, respectively [29].

With advancements in technology and the need for non-invasive cancer diagnostics, circulating miRNAs have gained significant attention as promising biomarkers for cancer detection. Due to their stability in extracellular biofluids, analytical accessibility, and high sensitivity, miRNAs are valuable indicators for disease diagnosis [49]. Numerous individual and combined miRNAs have been investigated for CRC screening, where certain miRNAs have been identified as strong candidates. Despite increased attention, currently there are no federally approved miRNA tests for colorectal cancer screening. However, a serum miRNA panel for detecting gastric cancer known as GASTROClear^®^ (Singapore) was approved by Singapore’s Health Sciences Authority in late 2019. This panel consists of 12 miRNAs with a sensitivity of 87.0%, a specificity of 68.4%, and an AUC of 0.92 in validation studies. This approval of a miRNA-based cancer screening test indicates a positive step towards the future adoption of similar tests for colorectal cancer. Therefore, this review aims to comprehensively study the latest findings in this matter. 

Many individual miRNAs have been analyzed as biomarkers for CRC detection, either as single markers or as part of panels consisting of multiple miRNAs. In this review, we found that the most commonly studied biomarker in the included articles was miR-21, appearing in both single and panel analyses across seven studies. This repeated investigation underscores the significance of miR-21 as a potential biomarker for colorectal cancer diagnosis and prognosis. The highest AUC of miR-21 as a single marker was 0.973 [12], while the lowest was 0.55 [39]. When combined with other markers, miR-21 showed varying AUC levels, ranging from 0.966 when combined with miRNA-18a [25], to 0.6385 when combined with miR-29a and miR-92a [17]. The differences in reported AUCs between these studies may be attributed to several factors. For instance, the [39] study involved a wider age range of patients compared to the [12] study. Additionally, [12] had a larger sample size in both healthy controls and CRC groups. Furthermore, the [39] study included more stage IV CRC patients (39%), whereas the [12] study had more patients in stages II (40%) and III (30%) with only 14% in stage IV. This might contribute to the differences in the reported AUCs of miR-21. The geographical element should not be ignored either; [12] conducted their study in Egypt among North African patients, while [39] conducted theirs in Germany, Europe. It has been reported that the ethnic group studied can influence the diagnostic capability of miRNAs [50].

It is worth mentioning that miRNA-21 has been a focus of many recent studies, concluding that it yields a high AUC and is highly accurate in diagnosing colorectal cancer. A recent meta-analysis confirmed the diagnostic potential of circulating miR-21 with a moderate sensitivity of 77% and a good specificity of 83% for CRC [51]. However, it was found that miR-21 has a low positive likelihood ratio (PLR < 10) and a high negative likelihood ratio (NLR > 0.1) which means CRC cannot be confirmed or ruled out. This point has to be considered when evaluating miRNA diagnostic metrics [52]. 

Another promising miRNA is miR-210, which is known for exhibiting oncogenic properties in various cancers, influencing cell proliferation; migration; invasion; and clonogenicity [53]. It has shown promising AUC and diagnostic metrics both as a single marker [12] or in a panel [19]. Among the analyzed biomarkers, miR-1246 arises as the microRNA displaying a high AUC 0.924 with 100 sensitivity and 80 specificity. The miR-1246 expression has been demonstrated as a potential diagnostic and prognostic biomarker for gastrointestinal cancers [54]. This miRNA is involved in regulation of multiple genes and signaling pathways that are involved in tumorigenesis; cell proliferation; angiogenesis; and metastasis [55]. A recent meta-analysis showed the robustness of circulating miR-1246 in different types of cancer, especially in breast cancer where they recommend it to be added to the already existing screening guidelines of breast cancer to improve diagnosis considering its stability and technical feasibility as a screening biomarker [56].

Recent studies indicate that miRNA panels generally offer greater sensitivity and specificity for CRC screening compared to individual miRNAs. Furthermore, there is generally no single miRNA associated with a tissue-specific condition; it is more a pattern expression condition, making it infeasible to use a single miRNA as a biomarker [57,58]. From this review, several miRNA panels have demonstrated promising AUCs. These panels vary in composition, containing two, three, four, five, or more miRNAs. Some studies reported improved diagnostic performance when miRNAs were combined with non-miRNA markers. For example, the combination of miR-211 and miR-25 with TGF-β1 yielded the highest reported AUC among the included studies compared to an AUC of 0.898 for the combination of miR-211 and miR-25 alone [29]. Another notable example is a panel of miR-15b-5p, miR-18a-5p, miR-29a-3p, miR-335-5p, miR-19a-3p, and miR-19b-3p, which showed an AUC of 0.75. This AUC was improved to 0.88 when combined with fecal occult blood testing, with a sensitivity of 81% and a specificity of 78% compared to 56% specificity when the miRNA panel was used alone [34].

Looking at the steadily rising incidence and mortality of early-onset colorectal cancer (EOCRC) compared to the decreasing trend in CRC-related mortality in adults 50 years or older, there is a pressing need to identify a non-invasive screening method to detect CRC among younger individuals who are not included in current CRC screening guidelines [59,60]. Due to the lack of screening programs for this age group, EOCRC is often detected at later stages with poor outcomes, underscoring the need for an early detection or a pre-screening test to determine which patients will benefit most from colonoscopies or any further interventions [61,62]. In this context, a highly specific test is favorable to identify those highly positive cases that warrant further investigation, such as a colonoscopy. A test with high specificity reduces the risk of false positives, thereby reducing the burden of further investigation and economic costs. A non-endoscopic test that is specific and sensitive for CRC, non-invasive, cost-effective, and generally acceptable to patients would be ideal. Unfortunately, this optimal tool for CRC screening does not yet exist. Currently FIT (fecal immunochemical test) is a non-invasive test used for this purpose with sensitivity ranges from 74 to 79% [63]. Scientist are working to find another non-invasive, non-endoscopic test that is sensitive and specific for CRC in young age groups in order to improve the diagnostic metrics and support current screening strategies [19].

Table 5 shows the miRNAs, both single and in panels, reported by studies that included patients below 50 years of age. It is worth mentioning that only one study specifically aimed to differentiate between miRNA signatures of early-onset colorectal cancer (EOCRC) and late-onset colorectal cancer (LOCRC) [19], which identified a panel of 4 miRNAs (miR-193a-5p, miR-210, miR-513a-5p, miR-628-3p) with an AUC of 0.92, a sensitivity of 90%, and a specificity of 80%. This panel was validated in another set of samples that maintained these metrics. Other studies included younger age groups within their samples but did not specifically study EOCRC versus LOCRC. The panel comprising miR-211, miR-25, and TGF-β1 showed the highest AUC of 0.993 and a specificity of 100%, which is a crucial metric for reducing false positives. If validated, this would lead to fewer further diagnostic tests and subsequently improve diagnosis accuracy.

Despite the advances in the understanding of the biological role of miRNAs and the promising results available, their translation into clinical practice is lagging. There are several technical, biological and environmental factors that complicate their adoption in the clinical setting. First of all, miRNA expression studies need to follow a standardized procedure and be followed by a validation study. Out of the 38 included articles in this review, 20 of them have used a validation step. As noted by [64], the miRNA panel evaluated by [19], while demonstrating promising results, has also exhibited significant diagnostic potential in non-cancer conditions such as septicemia and early-stage lung cancer. This underscores the need for a validation step using standardized methods to gain more precise insights. Furthermore, miR-21 is found to be dysregulated in different types of cancer such as prostate cancer, colorectal cancer and hepatocellular carcinoma [65]. This raises concerns about the organ specificity of miRNA-based CRC diagnostics. 

Furthermore, studies on miRNA should follow standard operating procedures (SOPs), which are crucial for transforming miRNA signatures into clinically meaningful tests. There is currently a lack of standardization in the processes and platforms used to quantify miRNA in clinical samples. Although almost all of the studies included in this review have used real-time quantitative reverse transcription PCR (qRT-PCR) for miRNA analysis, no information is available about the preanalytical and postanalytical sample processing. This makes it difficult to compare the results from different studies. To overcome this issue, a well-designed and a reproducible research protocol on normalization should be constructed to be used in studies that involve any cancer type and from any ethnic group. This protocol should consider steps starting from blood collection, plasma/serum preparation, storage, RNA extraction, and quantification [66]. This will ensure accurate interpretation and comparison of study results and the identification of miRNAs as specific and sensitive cancer biomarkers. Nevertheless, ethnic groups being studied have to be taken into consideration [67]. 

## 4. Conclusions and Limitations

This systematic review highlights the potential of circulating miRNAs as non-invasive biomarkers for early detection of colorectal cancer (CRC). Among the miRNAs investigated, miR-21 emerges as the most frequently studied and shows significant promise in both single-marker and panel-based analyses. However, the variability in diagnostic performance across studies underscores the need for standardization in miRNA quantification methods. The development and validation of miRNA panels, such as those combining multiple miRNAs or integrating non-miRNA markers, offer enhanced sensitivity and specificity particularly for detecting early-onset colorectal cancer (EOCRC). Despite the promising findings, the translation of miRNA biomarkers into clinical practice remains challenging due to technical, biological, and environmental factors. Standardized procedures, robust validation studies, and consideration of ethnic variability are essential for advancing the clinical utility of miRNAs in CRC screening. Future research should focus on well-designed, large-scale studies to establish reproducible and clinically relevant miRNA biomarkers that can complement existing screening strategies and improve early detection of CRC, especially in younger populations.

This systematic review has several limitations that should be considered. Firstly, the heterogeneity among included studies in terms of sample size, patient demographics, and CRC stages may affect the comparability of results. The lack of standardization in miRNA quantification methods across studies introduces variability and complicates the interpretation of findings. Additionally, the geographic and ethnic diversity of study populations may influence the expression profiles of miRNAs, limiting the generalizability of results. Although we identified promising miRNA panels, the validation of these panels in independent large-scale cohorts is necessary to confirm their diagnostic accuracy and clinical utility. Finally, publication bias could not be entirely ruled out, as studies with negative results are less likely to be published. Addressing these limitations through well-designed, standardized, and multi-centercenter studies will be crucial for the successful translation of miRNA biomarkers into clinical practice for CRC screening.

## 5. AI Tool Use Declaration

In preparing this manuscript, we employed the language model ChatGPT-4 (https://chatgpt.com/) accessed on 14 April 2024, developed by OpenAI, solely for the purpose of grammatical corrections and enhancing the readability of the text. The use of ChatGPT-4 was limited to editing assistance and did not influence the scientific content, data interpretation, or the conclusions drawn in this study.

## Figures and Tables

**Table 1:** Characteristics of the included studies.

Study	Year	Country of the Study	Study Design	Sample Size	Reported Age (Years)	EOCRC < 50	Sample Type	Method	Single/Panel	miRNA	AUC	Sensitivity	Specificity	Validation
[11]	2020	Egypt	Case-Control	HC = 48 CRC = 48	55.2 ± 7.9 Mean ± SD	Yes	Serum	qRT-PCR	Single	miR-21	0.94	95.80	91.70	No
[12]	2019	Egypt	Case-Control	HC = 101 CRA = 51 CRC = 35	51.97 ± 12.18 Mean ± SD	Yes	Serum	qRT-PCR	Single	miR-210	0.93	88.6	90.1	No
										miR-21	0.97	91.4	95.0	
										miR-126	0.67	88.6	50.5	
[13]	2019	Spain	Case-Control	HC = 100 CRA = 101 CRC = 96	65.7 ± 11.5 Mean ± SD	No	Plasma	qRT-PCR	Panel	miR-19a	0.95 = HC vs. CRC 0.91 = HC vs. CRA	91 = HC vs. CRC 95 = HC vs. CRA	90 = HC vs. CRC 90 = HC vs. CRA	5-fold cross validation
										miR-19b				
										miR-15b				
										miR-29a				
										miR-335				
										miR-18a				
[14]	2024	China	Case-Control	HC = 49 CRA = 51 CRC = 50	57.78 ± 9.75 Mean ± SD	Yes	Serum	qRT-PCR	Single	miR-22	0.74 = HC vs. CRC	-	-	No
											0.75 = HC vs. CRA	-	-	
[15]	2020	China	Case-Control	HC = 25 CRC = 25	59.7 ± 13.3 Mean ± SD	Yes	Serum	qRT-PCR	Panel	miR-203a-3p				Yes on 160 (80 CRC, 80 HC)
										miR-145-5p				
										miR-375-3p	0.89	81.25	73.33	
										miR-200c-3p				
[16]	2020	China	Case-Control	HC = 68 CRC = 148	Below and above 60	Not specified	Serum	qRT-PCR	Single	miR-92a-1	0.91	81.80	95.60	No
[17]	2020	Hawaii (USA) and Japan	Case-Control	HC = 18 CRC = 73	18 to 89 (Age range)	Yes	Serum	qRT-PCR	Single/Panel	miR-21	0.76 = miR-21	72.6	70.6	No
										miR-29a				
										miR-92a	0.64 = Combination of miR-21, miR-29a, and miR-92a	-	-	
[18]	2019	China	Case-Control	HC = 52 CRA = 22 CRC = 40	Below and above 55	Yes	Plasma	qRT-PCR	Single	miR-92b	0.79	-	-	No
[19]	2022	Japan and Spain	Case-Control	HC = 45 CRC = 72	44 (21–49) Mean (Age range)	Yes	Plasma	qRT-PCR	Panel	miR-193a-5p				Yes, (77 HC, 65 CRC)
										miR-210				
										miR-513a-5p	0.92	90	80	
										miR-628-3p				
[20]	2020	Egypt	Case-Control	HC = 45 CRC = 84	46.7± 10.3 Mean ± SD	Yes	Serum	qRT-PCR	Panel	let-7c miR-21 miR-26a miR-146a	0.95 = Combination (let-7c, miR-21, miR-26a and miR-146a)	91.8	91.7	90 subjects (60 CRC and 30 HC)
		
		
											0.95 = Combination of miR-21 and miR-26a	-	-	
[21]	2022	Iran	Case-Control	HC = 55 CRC = 45	38–67 Age range	Yes	Serum	qRT-PCR	Single	miR-1229,	0.81	-	-	In tissue
										miR-1246	0.84	-	-	
[22]	2020	Egypt	Case-Control	HC = 30 CRC = 37	49.1 ± 16.42 Mean ± SD	Yes	Serum	qRT-PCR	Single	miR-1246	0.92	100	80	No
										miR-451	0.76	73	80	
										miR-23a	0.67	-	-	
[23]	2020	Iran	Case-Control	HC = 45 CRC = 45	38–67 Age range	Yes	Serum	qRT-PCR	Single	miR-19a	0.87	-	-	No
										miR-20a	0.83	-	-	
										miR-150	0.75	-	-	
										miR-143	0.76	-	-	
										miR-145	0.78	-	-	
										let-7a	0.71	-	-	
[24]	2021	China	Case-Control	HC = 35 CRC = 100	Below and above 65	Not specified	Serum	qRT-PCR	Panel	miR-126 miR-1290 miR-23a miR-940				35 HCs and 100 CRC
		
											0.95	90	88.57	
		
[25]	2020	Egypt	Case-Control	HC = 50 CRC = 50	50.2 ± 16.7 Mean ± SD	Yes	Serum	qRT-PCR	Single/Panel	miR-18a	0.90	-	-	No
										miR-21	0.91	-	-	
										miR-92a	0.67	-	-	
											0.97 = Combined miRNA-18a + miRNA-21	-	-	
[26]	2020	China	Case-Control	HC = 20 CRC = 40	Below and above 60	Not specified	Serum	qRT-PCR	Single	miR-21	0.86	88.9	83.3	No
										miR-210	0.81	88.9	72.2	
[27]	2019	China	Case-Control	HC = 134 CRA = 20 CRC = 101	59.02 ± 13.2 Mean ± SD	Yes	Plasma	qRT-PCR	Panel	miR-144-3p				47 HC, 48 CRC
										miR-425-5p	0.95	93.80	91.30	
										miR-1260b				
[28]	2019	Iran	Case-Control	HC = 8 CRC = 12	58.7 Mean	Not specified	Serum	qRT-PCR	Single	miR-23a	0.89	-	-	13 CRC, 5 HC
										miR-301a	0.84	-	-	
[29]	2021	Egypt	Case-Control	HC = 40 CRC = 44	46.29 ± 10.0 Mean ± SD	Yes	Plasma	qRT-PCR	Panel	miR-211	0.89 = miR-211 + miR-25	82	90	
										miR-25	0.95 = miRNAs (211, 25, and 92a)	91	93	
										TGF-β1	0.99 = miRNAs (211, 25, and TGF-β1)	100	97	
[30]	2021	Germany	Case-Control	HC = 20 CRC = 20	64.8 ± 12.3 Mean ± SD	No	Serum	Next-generation sequencing (NGS)	Panel	let-7g-5p	0.80			198 CRC and 178 HC
										miR-19a-3p				
										miR-23a-3p				
										miR-92a-3p		-	-	
										miR-144-5p				
										miR-21-5p				
										miR-27a-3p				
[31]	2020	China	Case-Control	HC = 32 CRC = 32	62.22 ± 12.59 Mean ± SD	No	Serum	qRT-PCR	Panel	miR-30e-3p	0.731	-	-	80 CRC, 88 HC
										miR-31-5p	0.669	-	-	
										miR-34b-3p	0.785	-	-	
										miR-146a-5p	0.739	-	-	
										miR-148a-3p	0.648	-	-	
										miR-192-5p	0.652	-	-	
											0.883 = Three-miRNA Panel: miR-30e-3p, miR-146a-5p, and miR-148a-3p	80	79	
											0.932 = Six-miRNA Panel	85	86	
[32]	2023	China	Case-Control	HC = 40 CRC = 78	Below and above 60	Not specified	Plasma	qRT-PCR	Single/Panel	miR-126	0.73	-		Yes
										miR-139	0.82	-		
										miR-143	0.82	-		
										miR-595	0.86	-		
										miR-1237	0.67	-		
											0.95 = miR-126 + miR-139	-		
											0.94 = miR-126 + miR-139 + miR-143	-		
											0.95 = miR-126 + miR-139 + miR-595	-		
											0.95 = miR-126 + miR-139 + miR-595	-		
											0.90 = miR-126 + miR-139 + miR-143 + miR-595	-		
[33]	2022	China	Case-Control	HC = 30 CRA = 60 CRC = 60	66.95 ± 10.52 Mean ± SD	No	Serum		Single/Panel		HC vs. CRC	HC vs. CRC	HC vs. CRC	HC = 33, CRA = 33, CRC = 20
										miR-627-5p	0.97	87	100	
										miR-199a-5p	0.90	93	70	
										Combination (miR-627-5p + miR-199a-5p + CEA + CA19)	0.98	98	100	
											HC vs. CRA	HC vs. CRA	HC vs. CRA	
										miR-627-5p	0.84	84%	93%	
										miR-199a-5p	0.76	76%	53%	
										Combination (miR-627-5p + miR-199a-5p + CEA + CA19)	0.86	86%	77%	
[34]	2019	Spain	Case-Control	HC = 80 CRA = 74 CRC = 59	62.05 ± 54.3 Mean ± SD	No	Serum	qRT-PCR	Panel	miR-15b-5p miR-18a-5p miR-29a-3p miR-335-5p miR-19a-3p miR-19b-3p	0.74	81	56	
		
		
										Panel when combined with fecal HB				
										CRC vs. HC	0.88	81	78	
										HC vs. CRA	0.81	81	69	
[35]	2022	Japan	Case-Control	HC = 7 CRC = 7	58.4 ± 6.9 Mean ± SD	No	Serum	qRT-PCR	Single	miR-26a-5p	0.84	100	60	8 HC, 8 CRC
[36]	2020	China	Case-Control	HC = 50 CRC = 84	Below and above 60	Not specified	Serum		Single	miR-592	0.88	86.60	73.40	
[37]	2024	Turkey	Case-Control	HC = 20 CRC = 60	60 (31–81) Mean (Age range)	Yes	Serum	qRT-PCR	Single	let-7	0.76	70	70	
										miR-125b	0.76	70	65	
										miR-30a	0.939	93	75	
[38]	2021	China	Case-Control	HC = 150 CRC = 117	51.60 ± 11.41 Mean ± SD	Yes	Serum	qRT-PCR	Single/Panel	miR-15b	0.86	81.33	91.80	CRC = 80, HC = 67 HC
										miR-16	0.58	-	-	
										miR-21	0.75	95.06	94.44	
										miR-31	0.75	91.95	97.62	
											-	81.21 = miR-15b + miR-16 + miR-21	81.03 = miR-15b + miR-16 + miR-21	
											-	91.95 = miR-15b + miR-21 + miR-31		
[39]	2022	Germany	Case-Control	HC = 26 CRA = 20 CRC = 23	27–85 Age range	Yes	Serum	qRT-PCR	Panel	Let7	0.64	=	=	
										miR-16	0.67	-	-	
										miR-19	0.60	-	-	
										miR-21	0.55	-	-	
										miR-23	0.67	-	-	
										miR-29	0.64	-	-	
										miR-34	0.55	-	-	
										miR-92	0.64	-	-	
										miR-222	0.63	-	-	
										miR-451	0.65	-	-	
											0.90 = 10 miRs	65.2	95	
											0.87 = 5 miRs (miR-16, miR-19, miR-21, miR-34, miR-222)	59.5	95	
											0.80 = 3 miRNAs (miR-16, miR-19, miR-34	30.4	95	
											0.74 = 2 miRNAs (miR-16, miR-34)	21.7	95	
[40]	2022	China	Case-Control	HC = 34 CRC = 18	Below and above 65	Not specified	Serum	qRT-PCR	Panel	hsa-miR-3937	0.827	-	-	
											0.89 = (combined hsa-miR-3937 with CEA and CA199)	-	-	
											0.88 = (combined hsa-miR-3937 with CEA)	-	-	
											0.84 = (combined hsa-miR-3937 with CA199)	-	-	
[41]	2021	China	Case-Control	HC = 60 CRC = 164	32–75 Age range	Yes	Serum	qRT-PCR	Single	miR-192-5p	0.84	84.6	79.2	
[42]	2019	China	Case-Control	HC = 153 CRC = 165	Below and above 61	Not specified	Serum	qRT-PCR	Single	miR-99b-5p	0.63	32.1	90.8	Yes
										miR-150-5p	0.71	75.2	58.8	
[43]	2020	China	Case-Control	HC = 49 CRC = 51	58.92 ± 11.39 Mean ± SD	Yes	Serum	qRT-PCR	Single	miR-1539	0.67 (exosomal)	92 (exosomal)	40 (exosomal)	In tissues
											0.65 (serum)	38 (serum)	96.6 (serum)	
[44]	2020	China	Case-Control	HC = 90 CRC = 110	62.13 ± 9.21 Mean ± SD	No	Serum	qRT-PCR	Single/Panel	miR-378e	0.93	89	80	
											-	86 = (when combined with LI-cadherin)	94 = (when combined with LI-cadherin)	
[45]	2020	China	Case-Control	HC = 23 CRC = 80	66 (41~93) Mean (Age range)	Yes	Plasma	qRT-PCR	Single/Panel	miR-139-3p	0.73	-	-	Not mentioned
											0.87 = (When combined with CEA)			
[46]		China	Case-Control	HC = 76 CRC = 79	Below and above 60	Not specified	Plasma	qRT-PCR	Single	miR-103a-3p	0.76	-	-	Validation (30 CRC, 26 C)
										miR-127-3p	0.73	-	-	
										miR-151a-5p	0.74	-	-	
										miR-17-5p	0.74	-	-	
										miR-181a-5p	0.74	-	-	
										miR-18a-5p	0.78	-	-	
										miR-18b-5p	0.78	-	-	
[47]	2019	China	Case-Control	HC = 15 CRC = 15	Below and above 60	Not specified	Plasma	qRT-PCR	Single/Panel	miR-1290	0.96	78.79	93.33	CRC = 80, CRA = 50, HC = 30
										miR-320d	0.89	93.94	73.33	
											0.98 = Combined (miR-1290 + miR-320d)	90.91	93.33	
[48]	2021	Brazil	Case Control	HC = 27 CRA = 24 CRC = 41	Below and above 60	Not specified	Plasma	qRT-PCR	Panel	miR-28-3p let-7e-5p miR-106a-5p miR-542-5p	0.86	88.9	86.7	In tissues and in another dataset

**Table 2:** The highest and lowest reported AUC, sensitivity, and specificity for both single and panel markers in the included studies.

Measure	Single Marker		Combined Markers	
	Highest	Lowest	Highest	Lowest
AUC	(0.973)	(0.55)	(0.993)	(0.6385)
	miR-21	miR-21 = 0.55 and miR-34	miR-211 + miR-25 + TGF-β1	miR-21 + miR-29a + miR-92a
	[12]	[39]	[29]	[17]
Sensitivity	(100%)	(32.1%)	(100%)	(21.7%)
	miR-1246	miR-99b-5p	miR-211 + miR-25 + TGF-β1	miR-16 + miR-34
	[22]	[42]	[29]	[39]
Specificity	(100%)	(40%)	(100%)	(56%)
	miR-627-5p	Exosomal miR-1539	miR-627-5p + miR-199a-5p + CEA + CA19	miR-15b-5p + miR-18a-5p + miR-29a-3p + miR-335-5p + miR-19a-3p + miR-19b-3p
	[33]	[43]	[33]	[34]

**Table 3:** Single miRNAs with AUC above 0.7 with sensitivity and specificity values.

miRNA	AUC	Sensitivity	Specificity	Study
miR-21	0.973	91.4	95	[12]
	0.94	95.8	91.7	[11]
	0.918	84	96	[25]
	0.863	88.9	83.3	[26]
	0.75	95.06	94.44	[38]
miR-1290	0.96	78.79	93.33	[47]
miR-210	0.934	88.6	90.1	[12]
	0.818	88.9	72.2	[26]
miR-378e	0.9298	89	80	[44]
miR-1246	0.924	100	80	[22]
miR-92a-1	0.914	81.8	95.6	[16]
miR-320d	0.89	93.94	73.33	[47]
miR-15b	0.86	81.33	91.8	[38]
miR-192-5p	0.84	84.6	79.2	[41]
miR-150-5p	0.707	75.2	58.8	[42]

**Table 4:** miRNA panels with AUC above 0.7 with sensitivity and specificity values.

miRNA Panel	AUC	Sensitivity	Specificity	Study
miR-1290, miR-320d	0.98	90.91	93.33	[47]
miR-627-5p, miR-199a-5p, CEA, CA19	0.98	98	100	[33]
miR-211, miR-25, TGF-β1	0.993	100	97	[29]
miR-19a, miR-19b, miR-15b, miR-29a, miR-335, miR-18a	0.95	91	90	[13]
miR-126, miR-1290, miR-23a, miR-940	0.95	90	88.57	[24]
let-7c, miR-21, miR-26a, miR-146a	0.95	91.8	91.7	[20]
miR-144-3p, miR-425-5p, miR-1260b	0.954	93.8	91.3	[27]
miR-30e-3p, miR-31-5p, miR-34b-3p, miR-146a-5p, miR-148a-3p, miR-192-5p	0.932	85	86	[31]
miR-193a-5p, miR-210, miR-513a-5p, miR-628-3p	0.92	90	80	[19]
miR-211, miR-25, miR-92a	0.945	91	93	[29]
miR-211 + miR-25	0.898	82	90	[29]
miR-16, miR-19, miR-21, miR-34, miR-222	0.87	56.5	95	[39]
miR-30e-3p, miR-31-5p, miR-34b-3p, miR-146a-5p, miR-148a-3p, miR-192-5p	0.932	85	86	[31]
miR-15b-5p, miR-18a-5p, miR-29a-3p, miR-335-5p, miR-19a-3p, miR-19b-3p combined with fecal HB	0.88	81	78	[13]
miR-16, miR-19, miR-34	0.80	30.4	95	[39]
miR-15b-5p, miR-18a-5p, miR-29a-3p, miR-335-5p, miR-19a-3p, miR-19b-3p	0.74	81	56	[34]
miR-28-3p, let-7e-5p, miR-106a-5p, and miR-542-5p	0.86	88.9	86.7	[48]
miR-16, miR-34	0.74	21.7	95	[39]
miR-203a-3p, miR-145-5p, miR-375-3p, miR-200c-3p	0.893	81.25	73.33	[15]
let-7, miR-16, miR-19, miR-21, miR-23, miR-29, miR-34, miR-92, miR-222, miR-451	0.9	65.2	95	[39]
miR-19a, miR-19b, miR-15b, miR-29a, miR-335, miR-18a	0.95	91–95	90	[13]

**Table 5:** Performance of single and panel miRNA markers for EOCRC detection.

Single Markers				
miRNA	AUC	Sensitivity	Specificity	Study
miR-21	0.973	91.4	95	[12]
	0.94	95.8	91.7	[11]
	0.7558	72.6	70.6	[17]
Let-7	0.76	70	70	[37]
miR-210	0.934	88.6	90.1	[12]
miR-1246	0.924	100	80	[22]
miR-15b	0.86	81.33	91.8	[38]
miR-192-5p	0.84	84.6	79.2	[41]
**Panel Markers**				
**Panel**	**AUC**	**Sensitivity**	**Specificity**	**Study**
miR-211 + miR-25 + TGF-β1	0.993	97	100	[29]
miR-144-3p + miR-425-5p + miR-1260b	0.954	93.8	91.3	[27]
let-7c + miR-21 + miR-26a + miR-146a	0.95	91.8	91.7	[20]
miR-211 + miR-25 + miR-92a	0.945	91	93	[29]
miR-193a-5p + miR-210 + miR-513a-5p + miR-628-3p	0.92	90	80	[19]
Let7 + miR-16 + miR-19 + miR-21 + miR-23 + miR-29 + miR-34 + miR-92 + miR-222 + miR-451	0.90	65.2	95	[39]
miR-203a-3p + miR-145-5p + miR-375-3p + miR-200c-3p	0.893	81.25	73.33	[15]
miR-211 + miR-25	0.898	82	90	[29]
miR-16 + miR-19 + miR-21 + miR-34 + miR-222	0.87	56.5	95	[39]
miR-16 + miR-19 + miR-34	0.8	30.4	95	[39]
miR-16 + miR-34	0.74	21.5	95	[39]
